# Knockdown screening of chromatin binding and regulatory proteins in zebrafish identified Suz12b as a regulator of *tfpia* and an antithrombotic drug target

**DOI:** 10.1038/s41598-021-94715-2

**Published:** 2021-07-27

**Authors:** Revathi Raman, Weam Fallatah, Ayah Al Qaryoute, Sanchi Dhinoja, Pudur Jagadeeswaran

**Affiliations:** Department of Biological Sciences, University of North Texas, 1511 West Sycamore Street, Denton, TX 76203 USA

**Keywords:** Genetics, RNAi

## Abstract

Tissue factor pathway inhibitor (TFPI) is an anticoagulant protein that inhibits factor VIIa and Xa in the coagulation cascade. It has been shown that forkhead box P3 protein is a TFPI transcriptional repressor. However, there are no studies on chromatin remodeling that control TFPI expression. We hypothesized that the genome-wide knockdowns of the chromatin binding and regulatory proteins (CBRPs) in zebrafish could identify novel *tfpia* gene regulators. As an initial step, we selected 69 CBRP genes from the list of zebrafish thrombocyte-expressed genes. We then performed a 3-gene piggyback knockdown screen of these 69 genes, followed by quantification of *tfpia* mRNA levels. The results revealed that knockdown of *brd7*, *ing2*, *ing3*, *ing4*, and *suz12b* increased *tfpia* mRNA levels. The simultaneous knockdown of these 5 genes also increased *tfpia* mRNA levels. We also performed individual gene and simultaneous 5-gene knockdowns on the 5 genes in zebrafish larvae. We found that after laser injury, it took a longer time for the formation of the thrombus to occlude the caudal vessel compared to the control larvae. We then treated the larvae and adults with a chemical UNC6852 known to proteolytically degrade polycomb repressor complex 2, where SUZ12 is a member, and observed prolongation of time to occlude (TTO) the caudal vein after laser injury and increased *tfpia* mRNA levels in larvae and adults, respectively. In summary, our results have identified novel epigenetic regulators for *tfpia* and exploited this information to discover a drug that enhances *tfpia* mRNA levels and prolongation of TTO. This discovery provides the basis for testing whether UNC6852 could be used as an antithrombotic drug. This approach could be used to study the regulation of other plasma proteins, including coagulant and anticoagulant factors.

## Introduction

Tissue factor pathway inhibitor (TFPI) is an anticoagulant that inhibits blood coagulation pathway enzymes VIIa and Xa. This inhibition leads to reduced fibrin formation^1–4^. Thus, increasing the TFPI levels in plasma should be useful in controlling thrombosis. The TFPI levels are due to its cumulative expression and secretion from endothelial cells, megakaryocytes, monocytes, and smooth muscle cells^5–13^. However, limited information is available on the regulation of TFPI levels. For example, the *TFPI* gene promoter has been studied by transient expression assays in human endothelial cell lines. The C-polymorphism has been shown to be linked to increased expression of TFPI mRNA. The knockdown or overexpression of FOXP3 (forkhead box P3 protein) transcription factor resulted in an increase or decrease of TFPI expression, respectively, suggesting FOXP3 might be a repressor for TFPI expression^14^. It has also been shown that androgen-dependent TFPI regulating protein (ADTRP) controls TFPI gene transcription via POU1F1, a transcriptional activator^15^. Testosterone has been shown to upregulate TFPI expression in endothelial cells^16,17^. This upregulation has been shown to be dependent on reduced levels of a microRNA that is again regulated by testosterone^18^. Despite these studies, there are no approaches to alter the levels of endogenous TFPI via transcriptional activation directly. Such an approach would be valuable in controlling thrombosis. We hypothesized that modulating chromatin binding and regulatory proteins (CBRPs) might affect the TFPI levels and could be used as a drug target.

We used the zebrafish model to identify CBRPs that are involved in *tfpia* gene expression. We selected 69 CBRP genes from the single-cell RNAseq analysis of zebrafish thrombocyte transcripts identified in our laboratory, performed a knockdown screen of these genes in adult zebrafish, and measured *tfpia* mRNA levels by qRT-PCR. This screen revealed knockdown of *brd7*, *ing2*, *ing3*, *ing4*, and *suz12b* genes individually, increased *tfpia* mRNA levels. The simultaneous knockdown of these 5 genes in zebrafish larvae also showed prolongation of time to occlusion (TTO) of the caudal vein that is comparable to results of individual gene knockdowns. The treatment of the zebrafish adults and larvae with a SUZ12 inhibitor recapitulated the results of *suz12b* knockdown. In conclusion, our results showed knockdown of *brd7*, *ing2*, *ing3*, *ing4*, and *suz12b* increased *tfpia* mRNA levels, and SUZ12 inhibitor gave an antithrombotic phenotype. Thus, this epigenetic approach could be used to control coagulation factors.

## Materials and methods

All methods were carried out in accordance with University of North Texas guidelines and regulations. The study was carried out in compliance with the ARRIVE (Animal Research: Reporting of In Vivo Experiments) guidelines. All experiments with zebrafish were approved by the University of North Texas-Institutional Animal Care and Use Committee.

### Zebrafish aquaculture

Wild-type (WT) zebrafish were obtained from Ekkwill Tropical Fish Farm, Gibsonton, FL, and were maintained in the recirculating freshwater system at 28 °C (82 °F), pH 7.6 and supplemented with Instant Ocean. The fish were kept under a 14:10 h Light:Dark Cycle and were fed with live brine shrimp and fish flakes. One female and one male were placed in a breeding tank and separated by a divider for breeding. The following morning, the divider was removed once the lights get turned on. The embryos were transferred to embryonic E3 medium (5 mM NaCl, 0.17 mM KCl, 0.33 mM CaCl_2_, and 0.33 mM MgSO_4_, pH 7.2) in small plastic containers and the hatched larvae were used in further experiments.

### Piggyback knockdown and vivo morpholino (VMO) hybrid preparation

cDNA sequences for 69 CBRPs in zebrafish were obtained from Ensembl Genome Browser, and using primer 3 software, a gene-specific antisense oligonucleotide (ASO) within the coding sequence was designed. We divided these primers into 23 sets for 3-gene knockdowns. For each set of 3 mRNAs, 3 antisense oligonucleotides (ASOs) were designed (Supplementary Table S1). To these ASOs, at their 3′-ends, 3 oligonucleotides, 5′-TATAAAT-3′, 5′-TAACTGA-3′, and 5′-TAAGAGG-3′ were added respectively. For individual gene knockdowns, for each ASO, at its 3′-end, 5′-TATAAATTGTAACTG-3′ was added. The oligonucleotides were purchased from Invitrogen, Grand Island, NY. A VMO with a sequence 5′-CCTCTTACCTCAGTTACAATTTATA-3′ was purchased from Gene Tools LLC, Philomath, OR. For 3-gene knockdowns, the piggyback hybrid was prepared by mixing 2.5 μl of 0.5 mM VMO with 0.84 μl of each of 3 ASOs (1.5 mM of each ASO), and 0.5 μl of 10 × oligo-hybridization buffer (10 × OB) containing 500 mM NaCl, 10 mM Tris–HCl (pH 8.0), and 1 mM EDTA (pH 8.0) was added. For 5-gene knockdowns, the hybrid was prepared by mixing 2.5 μl of 1 mM VMO with 0.5 μl of each of 5 ASOs (2.5 mM of each ASO), and 0.5 μl of 10 × OB was added^19^. For individual gene knockdowns, the hybrid was prepared by mixing 2.5 μl of 0.5 mM VMO with 2.5 μl of 0.5 mM of ASO, and 0.5 μl of 10 × OB was added. The hybrid mixtures were heated to 94 °C for 5 min and slowly cooled to room temperature using a Takara PCR Thermal Cycler (Takara Bio, Mountain View, CA)^19^.

### Zebrafish injections

Adult zebrafish was placed on its lateral side on a clean paper towel, head covered with a wet Kimwipe. The skin was then gently wiped with a dry Kimwipe, and 5 μl of the above piggyback hybrid or 1 × PBS was then injected intravenously using a 27G1^1/4^ needle as described previously^19,20^. Three days post fertilization (dpf) larvae were injected with the above hybrid using micro-capillary injection needles (David Kopf Instruments, Tujunga, California, USA) prepared by a pipette puller. The needle tip was then clipped using forceps, loaded with 5 µl of either the above hybrid or 1 × PBS. The larvae were injected with 8 nl of the reagent intravenously using Picospritzer III (Parker Precision Fluidics, Hollis, NH) and a micromanipulator under an Olympus inverted microscope as described previously^21^. After injection, the larvae were transferred to E3 medium in a plastic container at 28 °C and incubated for 48 h.

UNC6852 (MedChem Express, Monmouth Junction, NJ), in 10% DMSO in saline were prepared at four different concentrations (0.075 mM, 0.15 mM, 0.3 mM and 0.6 mM). Eight nl of each of these solutions was injected into the 5 dpf larvae and incubated for 2 h. For controls, 10% DMSO in saline was injected. For adult fish injections, 5 µl of 0.6 mM UNC6852 was injected intravenously and incubated for 6 h.

### RNA extraction

Adult zebrafish were first anesthetized using 1 mM Tricaine in E3 medium. After 2 min, once the zebrafish flips on its side, it was removed from the medium, and an incision was made in the ventral surface from the anal pore up to the gills using a pair of scissors. The visible liver and spleen were retrieved using a pair of forceps. To this 15 mg tissue, 150 µl of TRI Reagent (Sigma-Aldrich, St. Louis, MO) was added, and homogenized using PRO200 MULTI-GEN 7XL Homogenizer (PRO Scientific Inc., Oxford, CT) for 1 min. After incubating the homogenate for 10 min, 15 µl of 1-bromo-3-chloropropane was added and centrifuged for 15 min at 10,000 × *g*. The aqueous phase was transferred into another tube, and 200 µl of isopropanol was added. After incubating for 10 min, the sample was centrifuged again. The pellet obtained was finally washed with 75% ethanol and suspended in nuclease-free water, and the RNA was stored at − 80 °C for future use.

### Quantitative real-time PCR (qRT-PCR)

To 1 µl of the liver and spleen RNA (1 µg/µl), 4 µl of qScript cDNA SuperMix (Quanta Bio, Beverly, MA) and 15 µl of nuclease-free water were added. This mixture was subjected to the following cycles, 25 °C for 5 min, 42 °C for 40 min, and 85 °C for 5 min, and finally held at 4 °C to generate cDNA. One µl of this cDNA was amplified using 1 µl of 25 µM of each of the qRT-PCR *tfpia* primers (forward primer: 5′-CTCCCAACCAGCTAAACAGG-3′ and reverse primer: 5′-GCGAAAGACTTGACATCTGC-3′) and 1 drop of 1-Drop PCR Mix. For control experiments, we used *β-actin* primers (forward primer: 5′-TCTCTTGCTCCTTCCACCAT-3′ and reverse primer: 5′-CATCGTACTCCTGCTTGCTG-3′). One µl of the amplified PCR products (cDNA) was checked using 1.2% agarose gel electrophoresis. Subsequently, 1 µl of the above cDNA sample was mixed with 5 µl of PowerUp SYBR Green MasterMix (Thermo Fisher Scientific, Grand Island, NY), 0.2 µl of each of the qRT-PCR primers, and 3.6 µl of nuclease-free water. qRT-PCR was performed using this mixture for 45 cycles (ViiA 7 by Life Technologies, Applied Biosystems, Grand Island, NY). The data were collected for 6 individual WT samples as duplicates for their Ct values for both *β-actin* and *tfpia* gene expression, and the average of the duplicates was used as Ct values for each WT sample. The ΔCt values for WT samples were calculated as a difference between Ct values of *β-actin* and *tfpia*. The average ΔCt value for WT was then calculated. Subsequently, from each ΔCt value, the average ΔCt value was subtracted, and the resulting ΔΔCt value was used to calculate fold change for each of the 6 individual samples using the formula 2^−ΔΔCt^, representing the change in *tfpia* gene expression relative to *β-actin.* Similarly, in the knockdown samples, from each ΔCt value, the average ΔCt value of the WT was subtracted, and the resulting ΔΔCt value was calculated, followed by the calculation of fold change.

### Laser-induced venous thrombosis

For laser-induced venous thrombosis, 5 dpf larvae were transferred into a 1.5 ml Eppendorf tube containing 0.5 ml E3 medium followed by the addition of 10 µl of 10 mM MS222. After 2 min of anesthesia, 0.5 ml of 1.6% low melting agarose at 37 °C was added to the Eppendorf tube, and the contents were mixed gently by pipetting up and down using a transfer pipette. The contents were then poured along with the larvae into a chamber made using the rectangular rubber gasket by pressing it on to a thin coat of petroleum jelly on a microscopic slide. The larvae were adjusted with a pipette tip such that they were lying on their lateral sides. The slide with the larvae was focused with a 20 × objective of the Nikon Optiphot fluorescence microscope. The pulsed nitrogen laser with a wavelength of 445 nm, routed through the coumarin-440 dye (Micro Point Laser, Stanford Research Systems Inc., Sunnyvale, CA), was delivered at 10 hits per cycle through the fluorescence port to hit the mid-region in the caudal vein located in the 5th somite posterior to the anal pore^22^. TTO of the vessel was measured as the time taken to occlude the vessel from the time the laser hits the vessel until the vessel completely occludes. If the vessel did not occlude until 60 s, TTO was recorded as 60 s.

### Statistical analysis

Statistical analysis was performed using GraphPad Prism version 9.0.0, GraphPad Software, San Diego, California, USA. Statistical significance was assessed by one-way ANOVA followed by Dunnett’s multiple comparison test or Student’s *t* test. p-value < 0.05 was considered significant.

## Results

**Figure 1:**
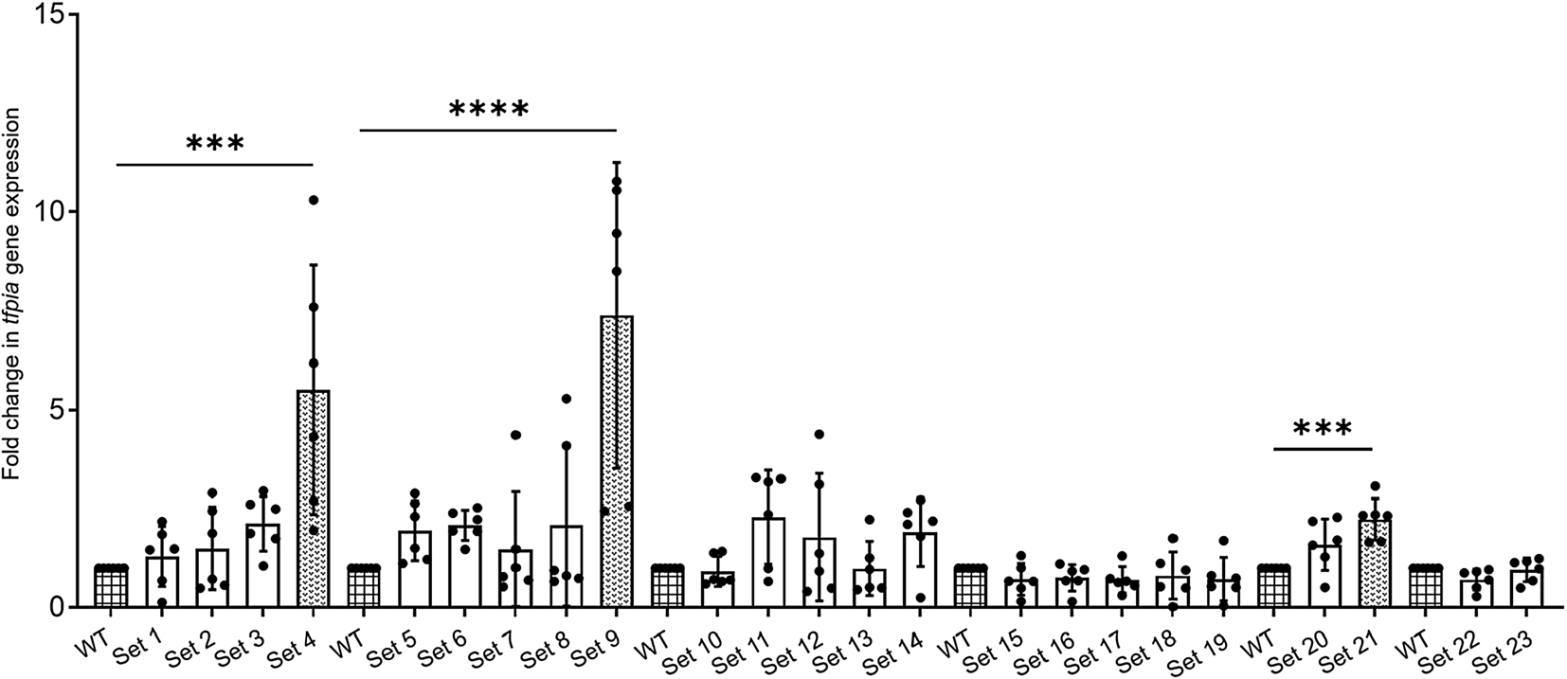
Levels of *tfpia* mRNA in a primary knockdown screen of chromatin binding and regulatory proteins. Quantitative real-time PCR showing the fold change of *tfpia* gene expression in liver and spleen in 23 sets (Set 1–Set 23) of chromatin binding and regulatory protein-knockdowns compared to daily wild-type (WT) controls using one-way ANOVA. The bar graphs represent daily wild-type controls (squared bars), and all knockdowns (open bars and dotted bars) to the right of each wild-type were performed on the same day. Error bars represent mean ± SD. Six fish were used for each set of chromatin binding and regulatory protein-knockdown and control experiments (N = 6). The lines on the top represent a significant difference between wild-type and knockdown sample sets. *** and **** represent p ≤ 0.001 and p ≤ 0.0001, respectively. p-value < 0.05 was considered significant.

**Figure 2:**
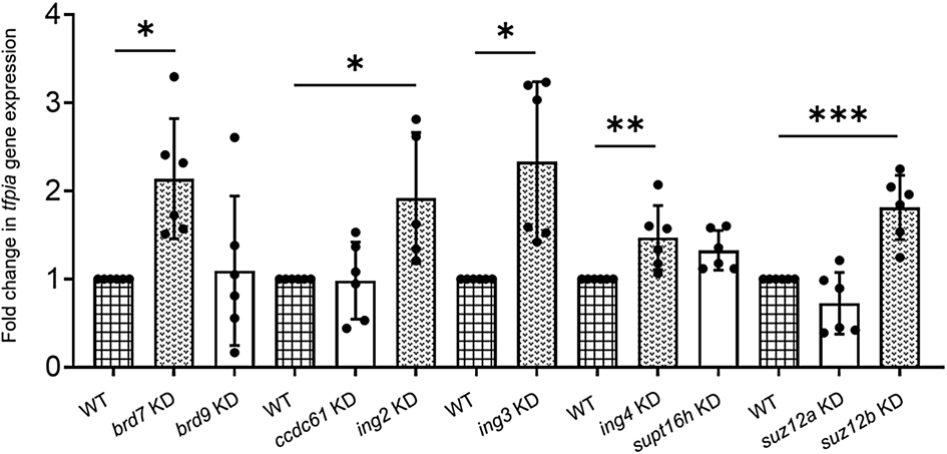
Levels of *tfpia* mRNA in a secondary knockdown screen of chromatin binding and regulatory proteins. Quantitative real-time PCR showing the fold change of *tfpia* gene expression in liver and spleen in knockdown of 9 chromatin binding and regulatory protein genes, *brd7*, *brd9*, *ccdc61*, *ing2*, *ing3, ing4, supt16h, suz12a,* and *suz12b*, with daily wild-type (WT) controls using one-way ANOVA. The bar graphs represent daily wild-type controls (squared bars), and all knockdowns (open bars and dotted bars) to the right of each wild-type were performed on the same day. Error bars represent mean ± SD. Six fish were used for each of the chromatin binding and regulatory protein gene knockdown and control experiments (N = 6). The lines on the top represent a significant difference between wild-type and knockdown samples. *, **, and *** represent p ≤ 0.05, p ≤ 0.01, and p ≤ 0.001, respectively. p-value < 0.05 was considered significant.

Since TFPI is expressed in megakaryocytes and thrombocytes have similar transcription factors expressed in megakaryocytes, we first examined single-cell RNAseq data from our laboratory for the transcripts expressed in zebrafish thrombocytes (manuscript in preparation) and found transcripts for 69 CBRPs as well as *tfpia* transcripts. To identify whether any of the 69 CBRPs are involved in *tfpia* gene expression, as an initial step, we selected all 69 CBRP mRNAs, and they were divided into 23 sets for a 3-gene knockdown. For each set of 3 mRNAs, 3 ASOs were designed with 3 different heptamer sequences at their 3′-ends, such that these heptamer sequences will form hybrids with a VMO. We then hybridized each set of 3 ASOs with one VMO and injected the piggyback hybrid into the zebrafish intravenously. PBS-injected WT was used as a control. We used 6 adult zebrafish for each of the WT control and experimental set. Forty-eight hours later, RNA was isolated from the liver and spleen of each of the WT and piggyback hybrid-injected fish. This RNA was subjected to qRT-PCR using *tfpia* primers to check for the *tfpia* gene expression levels. *β-actin* was used as an internal control. In our primary screening, we identified that knockdown of 3 sets of mRNAs (Set 4, Set 9, and Set 21), corresponding to 9 individual genes, *brd7*, *brd9*, *ccdc61*, *ing2*, *ing3*, *ing4*, *supt16h*, *suz12a*, and *suz12b*, showed an increase in *tfpia* expression (Fig. 1). In our secondary screen, 9 genes from the above 3 sets were subjected to individual gene knockdowns and checked for *tfpia* gene expression using qRT-PCR. Out of 9 genes, 5 genes, *brd7*, *ing2*, *ing3*, *ing4,* and *suz12b*, after knockdown, showed an increase in *tfpia* mRNA levels (Fig. 2).

**Figure 3:**
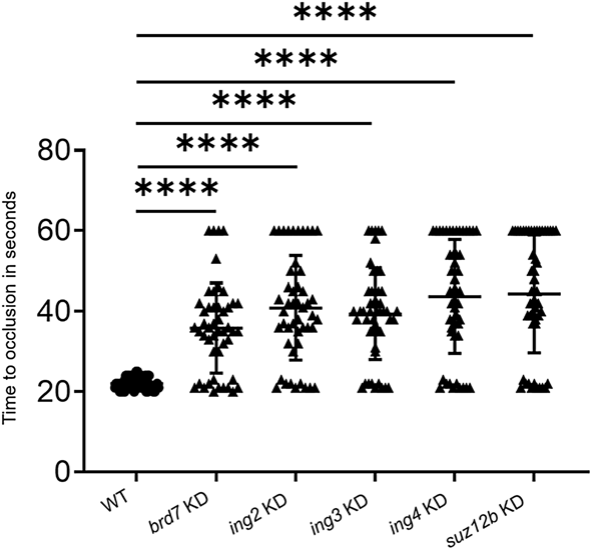
Comparison of time to occlusion of the caudal vein after laser injury between wild-type control 5 dpf larvae and *brd7*, *ing2*, *ing3*, *ing4*, and *suz12b* knockdown larvae by one-way ANOVA. The dot plots represent wild-type larvae (closed circles) and the knockdown larvae (closed triangles). Error bars represent mean ± SD. p-value < 0.05 was considered significant, and 50 larvae each were used in control and knockdown experiments (N = 50). The lines on the top represent a significant difference between wild-type and knockdown samples. **** represents p ≤ 0.0001.

To confirm the adult knockdown results in larvae, we injected the piggyback hybrid for each of the above 5 genes individually into 3 dpf larvae. PBS-injected 3 dpf WT larvae were used as a control. We used 50 zebrafish larvae for each of the WT control and experimental set. Forty-eight hours post knockdown, laser-induced venous thrombosis was performed on 5 dpf larvae. The piggyback hybrid-injected larvae showed prolonged TTO compared to PBS-injected WT control larvae (Fig. 3).

**Figure 4:**
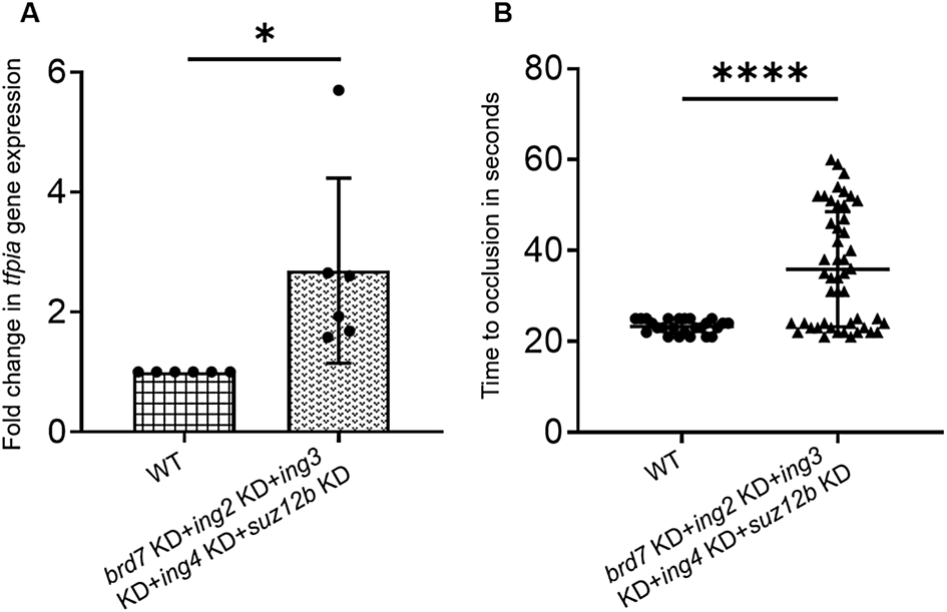
Simultaneous 5-gene knockdowns in adults and larvae. (**A**) Quantitative real-time PCR showing the fold change of *tfpia* gene expression in liver and spleen in simultaneous knockdown of 5 chromatin binding and regulatory protein genes, (*brd7* + *ing2* + *ing3* + *ing4* + *suz12b*) with wild-type (WT) controls using Student’s *t* test. The bar graphs represent wild-type control (squared bar) and simultaneous 5-gene knockdown (dotted bar). Error bars represent mean ± SD. Six fish were used for each of the simultaneous 5-gene knockdown and control experiments (N = 6); (**B**) Comparison of time to occlusion of the caudal vein after laser injury between wild-type control 5 dpf larvae and simultaneous 5-gene knockdown larvae (*brd7* + *ing2* + *ing3* + *ing4* + *suz12b*) by Student’s *t* test. The dot plots represent wild-type larvae (closed circles) and the knockdown larvae (closed triangles). Error bars represent mean ± SD. For experiments in (**A**) and (**B**), p-value < 0.05 was considered significant, and 50 larvae each were used in control and knockdown experiments (N = 50). The lines on the top represent a significant difference between wild-type and knockdown samples. * and **** represent p ≤ 0.05 and p ≤ 0.0001, respectively.

To examine the effects of simultaneous knockdown of all these 5 genes, we prepared 2 sets of piggyback hybrids with 3 ASOs in one set and 2 ASOs on the other set. We then mixed these 2 sets of 5 ASOs with one VMO (2 × concentration) to prepare the final piggyback hybrid of 5 ASOs and used that in simultaneous 5-gene (*brd7* + *ing2* + *ing3* + *ing4* + *suz12b*) knockdown experiments. The results showed increased *tfpia* gene expression in the above 5-gene knockdowns (Fig. 4a). We also performed laser-induced thrombosis after simultaneous knockdown of all 5 genes together (*brd7* + *ing2* + *ing3* + *ing4* + *suz12b*). The results showed a prolonged TTO as seen in individual gene knockdown experiments (Fig. 4b).

**Figure 5:**
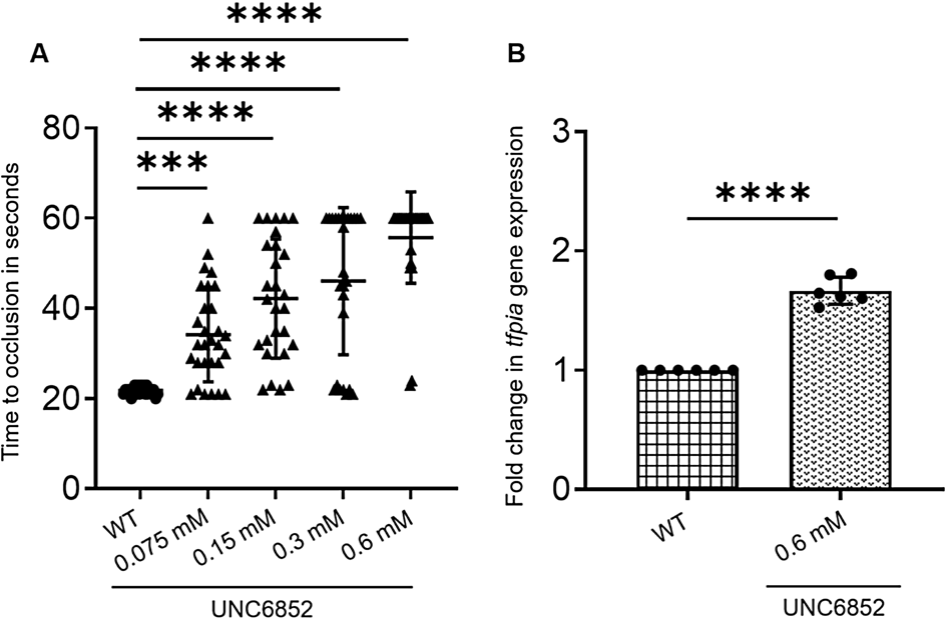
Treatment of zebrafish larvae and adult with a PRC2 inhibitor, UNC6852. (**A**) Comparison of time to occlusion of the caudal vein after laser injury between wild-type control 5 dpf larvae and UNC6852-injected larvae by one-way ANOVA. The x-axis represents four different concentrations of the UNC6852 (0.075 mM, 0.15 mM, 0.3 mM and 0.6 mM) used for larval injections. The dot plots represent wild-type larvae (closed circles) and the knockdown larvae (closed triangles). Error bars represent mean ± SD. 50 larvae each were used in control and UNC6852 injections (N = 50); (**B**) Quantitative real-time PCR showing the fold change of *tfpia* gene expression in liver and spleen for UNC6852-injected adult zebrafish with wild-type control by Student’s *t* test. The concentration of UNC6852 used for adult injections (0.6 mM) is indicated beneath the bar graph. Error bars represent mean ± SD. Six fish were used for each of the simultaneous 5-gene knockdown and control experiments (N = 6). *** and **** represent p ≤ 0.001 and p ≤ 0.0001, respectively.

To check whether we can inhibit CBRPs encoded by the 5 genes identified above, we searched for commercially available chemical inhibitors for these proteins. We found UNC6852 as an inhibitor for polycomb repressor complex 2 (PRC2), where SUZ12 is a member. To test whether UNC6852 inhibits PRC2 and yields results similar to the knockdown of *suz12b*, we injected intravenously 5 dpf larvae with 4 different concentrations of the UNC6852. After 2 h, we subjected these larvae to laser-induced venous thrombosis. We found a dose-dependent prolongation of TTO in the above larvae (Fig. 5a) compared to control larvae and also found the maximal prolongation of TTO was at 0.6 mM of UNC6852. To test whether this inhibitor enhances *tfpia* mRNA levels, we injected the adult fish with 5 µl of 0.6 mM of UNC6852 and found increased *tfpia* mRNA levels 6 h post-injection (Fig. 5b).

## Discussion

In this study, we performed simultaneous 3-gene knockdowns of 69 genes encoding CBRPs and tested their effects on *tfpia* gene expression. We found 5 genes when they are knocked down individually, resulted in increased *tfpia* mRNA levels. qRT-PCR was chosen as the assay for identifying regulators because it is a sensitive assay for studying mRNA levels. Since there is internal control, this assay to monitor *tfpia* levels is robust and reliable. Thus, the use of qRT-PCR to screen 23 sets of genes was relatively easy. *brd7*, *ing2*, *ing3*, *ing4,* and *suz12b* genes were identified in our screen as regulatory genes controlling the *tfpia*. Since we only studied 69 CBRP genes from the list of thrombocyte-expressed genes and did not include all the CBRPs in the zebrafish genome, it appears random, and thus, there is a chance that we may not discover all the CBRPs involved in *tfpia* gene regulation. This is the major caveat of this study. Future knockdown screening of all CBRP genes in the zebrafish genome is needed to discover the novel regulators of the *tfpia* gene comprehensively.

BRD7 is a member of the bromodomain-containing protein family, which plays an important role in chromatin remodeling, transcriptional regulation, and cell signaling. BRD7 both positively and negatively regulates the transcription of a number of genes such as ERα and the androgen receptor genes, respectively^23,24^. Since ERα decreases the transcription of *TFPIα* in humans and Brd7 in zebrafish regulates Erα, it is possible that Brd7 indirectly regulates *tfpia*. In addition, human BRD7 has been shown to participate in chromatin remodeling^25^. Our results show that *tfpia* transcription is enhanced when *brd7* transcripts are reduced. Thus, we hypothesize that Brd7 appears to be a repressor for *tfpia* gene transcription.

The ING (inhibitor of growth) family of proteins in humans are classified as tumor suppressors involving transcriptional regulation of genes through the p53 pathway and chromatin regulation^26^. Loss of these factors could lead to cellular senescence and cancer involving mechanism such as cell proliferation, apoptosis, and DNA repair^26,27^. Our observation that the knockdown of *ing2*, *ing3*, and *ing4* resulted in an increase in *tfpia* gene transcription suggests that the ING family of proteins are also repressors for *tfpia* gene and probably involves chromatin regulation.

SUZ12 (Suppressor of Zeste 12) is a member of PRC2, which consists of 2 additional proteins, a non-enzymatic EED (Embryonic Ectoderm Development) and a methyltransferase EZH2 (Enhancer of Zeste Homolog 2). The PRC2 complex is responsible for the methylation of histone H3 resulting in gene repression^28^. Loss of Suz12 in mice led to loss of H3 methylation and resulted in increased expression of Hoxa cluster genes^29^. Our results showed knockdown of *suz12b* resulted in increased *tfpia* expression. These results are also in line with our observations of enhanced transcription of *tfpia* in the presence of UNC6852, a SUZ12 inhibitor^30^.

Surprisingly, the simultaneous knockdown of these 5 genes did not result in a greater expression of *tfpia* than that shown by individual gene knockdowns. This is possible because the individual gene knockdowns might have already reached a threshold for *tfpia* expression. For example, Brd7, Ing family of proteins, and Suz12 appear to be repressors for *tfpia* gene transcription. Thus, since the loss of each of these three types of regulators is involved in the repression phenotype reversal through different mechanisms, the knockdown of all these regulators together may not result in enhanced loss of repression.

Even though the knockdown of the above 5 repressors resulted in increased *tfpia* mRNA levels, we cannot rule out whether these knockdowns are directly affecting *tfpia* gene expression or whether they have an indirect effect via the expression of an activator for *tfpia* gene promoter. Future studies should resolve this issue.

Our earlier studies have shown that the average efficiency of the 3-gene knockdown method is 85–90%^19^. Thus, even though there is this variability since, in all cases, it has been > 85%, we believe the knockdowns of CBRPs should follow similar efficiencies. In the worst-case scenario, there is no knockdown at all, or the efficiency of knockdown is < 85%. In such situations, we might miss identifying a target gene. In fact, this problem is inherent to the screening procedures. However, when the positives are identified at the protein level indirectly by the functional evaluation, such as prolongation of TTO, they are invariably due to high knockdown efficiencies. In this study, we did not demonstrate the change in Tfpia levels as a function of the change in transcription of *tfpia*. It must be noted that even with human plasma, it has been very difficult to identify TFPI with antibodies. Thus, with only a limited amount of plasma that is obtained from zebrafish, it is difficult to show the changes of Tfpia expression at the protein level. The development of more sensitive methods to detect Tfpia may resolve this issue. Since it was not possible to study the Tfpia knockdown at the protein level, the vascular occlusion differences could not be due to changes in Tfpia levels alone, and it is possible that the differences in vascular occlusion could be due to changes in other coagulation factor levels as well. This remains to be studied in the future.

Since TFPI is produced from megakaryocytes, endothelium, monocytes, and smooth muscle cells, our transcription analysis may represent regulation of *tfpia* from all these cells. This is possible because we used RNA from the liver and spleen that carry all the above cell types.

Earlier studies have been shown that UNC6852 is a chemical degrader for PRC2 complex affecting SUZ12 and thereby affecting methyltransferase activity^30^. Our results showing increased transcription of *tfpia* in adult zebrafish and also prolonged TTO in the larvae when their blood vessels were injured by the laser, in the presence of UNC6852 are consistent with our knockdown data of *suz12b*. This is because increased levels of *tfpia* may increase the anticoagulant activity and thus, lead to prolonged TTO. Unfortunately, in larvae we could not isolate liver and spleen to prepare RNA for quantifying *tfpia* transcription. The above results provide the basis for using drugs like UNC6852 that control *tfpia* in an epigenetic manner and offer a novel antithrombotic target. Interestingly, estrogen-treated individuals, patients with breast cancer and lung cancer have reduced levels of TFPI and exhibited venous thrombosis^31–34^. Thus, the above drug target would be a viable alternative to prevent thrombotic episodes in these patients.

In summary, our results revealed that proteins encoded by *brd7*, *ing2*, *ing3*, *ing4*, and *suz12b* are negatively regulating *tfpia* gene transcription and the reduction in their gene expression led to increased *tfpia* mRNA levels. Also, it led to prolonged clotting time and thus, giving a bleeding tendency. UNC6852 also produced this bleeding phenotype. Thus, our approach could be used to design novel proteolytic drugs that enhance TFPI levels via inhibiting the epigenetic proteins in treating patients with thrombotic risk. Moreover, our approach could be used to study the regulation of other proteins including coagulation factors and other plasma proteins.

## Supplementary Information

Supplementary Table S1.
