# Supplementary material for: Knockdown screening of chromatin binding and regulatory proteins in zebrafish identified Suz12b as a regulator of tfpia and an antithrombotic drug target
Source: Sci Rep. 2021 Jul 27;11:15238. doi: 10.1038/s41598-021-94715-2 (PMC8316476; doi:10.1038/s41598-021-94715-2)
Supplement: Supplementary file 1 — Supplementary Table S1. [file 41598_2021_94715_MOESM1_ESM.docx]

Supplementary Table S1: List of genes and the antisense primers used in the knockdown screen. In the 23 sets, each set has three genes grouped together. The antisense primer sequence is shown in black, whereas the sequences shown in different font colors are involved in hybrid formation with VMO. The antisense primer sequences shown in bold font in the primary screen are also used in the secondary screen, except that these primers had TATAAATTGTAACTG at their 3’ ends.

| **Gene Sets** | **Ensembl ID** | **Gene Description** | **Gene Name** | **ASOs (5'-3') for Primary Screen** |
| --- | --- | --- | --- | --- |
| Set 1 | ENSDARG00000025467 | apoptosis antagonizing transcription factor | aatf | GCTCTTGAACTCTGAGAATGTTTGTTATAAAT |
|  | ENSDARG00000006487 | acidic (leucine-rich) nuclear phosphoprotein 32 family, member A | anp32a | AACATTTTCTCTGTAGTCGTTCAGGTAACTGA |
|  | ENSDARG00000023330 | acidic (leucine-rich) nuclear phosphoprotein 32 family, member B | anp32b | ACTTCACAATTGAAAAGGTCAAGACTAAGAGG |
| Set 2 | ENSDARG00000054804 | acidic (leucine-rich) nuclear phosphoprotein 32 family, member E | anp32e | CTTGTTGCCACTCAAATTAAGGTAGTATAAAT |
|  | ENSDARG00000101037 | anti-silencing function 1Ba histone chaperone | asf1ba | GAAGCTCAGTGTCCGTATACTCATTTAACTGA |
|  | ENSDARG00000043713 | anti-silencing function 1Bb histone chaperone | asf1bb | CTCATTGTTCACATAGTAGCCAATGTAAGAGG |
| Set 3 | ENSDARG00000099274 | ataxin 3 | atxn3 | CAAATATAGAGTAACCCTCCTGCTGTATAAAT |
|  | ENSDARG00000037009 | barrier to autointegration factor 1 | banf1 | AACAACTCCTCATCTTTCCTCAGTATAACTGA |
|  | ENSDARG00000102974 | bromodomain adjacent to zinc finger domain, 2A | baz2a | ATTCTTGCCTTTCTCTTCATTTTCTTAAGAGG |
| Set 4 | ENSDARG00000008380 | bromodomain containing 7 | brd7 | **TTAAACCATCAGATGTCCTTCTCTC**TATAAAT |
|  | ENSDARG00000017143 | bromodomain containing 9 | brd9 | **ATATCACTGATGATGGGCTCTTTTA**TAACTGA |
|  | ENSDARG00000062307 | coiled-coil domain containing 61 | ccdc61 | **CTAAGTTCCTCTAACTGTTCCACCA**TAAGAGG |
| Set 5 | ENSDARG00000062152 | chromatin assembly factor 1, subunit A (p150) | chaf1a | GTCCTCTAGCACAGAGTCTTCACATTATAAAT |
|  | ENSDARG00000056473 | chromatin assembly factor 1, subunit B | chaf1b | AGTCGATATGGCAAATTAAATGTGTTAACTGA |
|  | ENSDARG00000070108 | DEK proto-oncogene | dek | GGGTTTGGACTTTCCACTTACTACTTAAGAGG |
| Set 6 | ENSDARG00000042087 | DNA methyltransferase 1 associated protein 1 | dmap1 | TTAGTTTGCCACAAATGCAGTAATATATAAAT |
|  | ENSDARG00000099640 | embryonic ectoderm development | eed | ATGACCAACATAATGCTTCACACACTAACTGA |
|  | ENSDARG00000054793 | ER membrane protein complex subunit 10 | emc10 | TGGTTTTGTGAGAGATACACACTGTTAAGAGG |
| Set 7 | ENSDARG00000060054 | enhancer of polycomb homolog 1 (Drosophila) b | epc1b | AGAGTGATATTGACAGCCTGTTCTCTATAAAT |
|  | ENSDARG00000007485 | enhancer of polycomb homolog 2 (Drosophila) | epc2 | TGCTCTTTTCTCTTTTCTTGATCATTAACTGA |
|  | ENSDARG00000079020 | GC-rich sequence DNA-binding factor 2 | gcfc2 | ACACCAATACTAACTACTGGAAGCGTAAGAGG |
| Set 8 | ENSDARG00000099572 | high mobility group nucleosomal binding domain 2 | hmgn2 | CTTCTCACCATCAATCTTTCTTTTGTATAAAT |
|  | ENSDARG00000036754 | high mobility group nucleosomal binding domain 3 | hmgn3 | ATCTTTCTCCTCTTTCTTTCCTTTGTAACTGA |
|  | ENSDARG00000019116 | inhibitor of growth family, member 1 | ing1 | CAGTATATCAGAAATCGTCGTCATGTAAGAGG |
| Set 9 | ENSDARG00000104907 | inhibitor of growth family, member 2 | ing2 | **CCCACTGTCACTCTCCTTTTTATAC**TATAAAT |
|  | ENSDARG00000034326 | inhibitor of growth family, member 3 | ing3 | **AACTTCTTTTCAGGTACGTGTTCAG**TAACTGA |
|  | ENSDARG00000030716 | inhibitor of growth family, member 4 | ing4 | **GAGGTAGAGTCATAATCGGTGCTTT**TAAGAGG |
| Set 10 | ENSDARG00000022413 | inhibitor of growth family, member 5a | ing5a | TTCTGTTATCTGGACTCTCATAGCCTATAAAT |
|  | ENSDARG00000068175 | inhibitor of growth family, member 5b | ing5b | ATCCACCATTTCATATATCTGCATTTAACTGA |
|  | ENSDARG00000059794 | lysine (K)-specific demethylase 6A, like | kdm6al | CTTGTGCAGTTGTGAGGTAGTGTGTTAAGAGG |
| Set 11 | ENSDARG00000056929 | lysine (K)-specific demethylase 6B, b | kdm6bb | TGTGTATTAAGAAGAGAGGGATTGGTATAAAT |
|  | ENSDARG00000033443 | MDM2 proto-oncogene | mdm2 | TCTGCGAAGATTGTGACTTCATATATAACTGA |
|  | ENSDARG00000057513 | MDM4 regulator of p53 | mdm4 | GTAATTACTTCGCAAGTTACCCAAATAAGAGG |
| Set 12 | ENSDARG00000041155 | mortality factor 4 like 1 | morf4l1 | CCATGGTTTTAATTCTTCAGGAATTTATAAAT |
|  | ENSDARG00000078214 | MYB binding protein (P160) 1a | mybbp1a | TTCTTCTTTTTGGATTTCTTAGCCTTAACTGA |
|  | ENSDARG00000034693 | Myb-like, SWIRM and MPN domains 1 | mysm1 | CAGCTGATTCACACTTATCTGCTAATAAGAGG |
| Set 13 | ENSDARG00000101813 | nucleosome assembly protein 1-like 1 | nap1l1 | TTAATGTCTTGTAAGTGCTTCAGGATATAAAT |
|  | ENSDARG00000070560 | nucleosome assembly protein 1-like 4a | nap1l4a | TTTAAAGATTGTAAGCCAGAAATCGTAACTGA |
|  | ENSDARG00000068868 | nucleosome assembly protein 1-like 4b | nap1l4b | AATTCCCTTAGGTTTCTCCTCATTATAAGAGG |
| Set 14 | ENSDARG00000035285 | nuclear receptor corepressor 1 | ncor1 | CATACTGAGCTCTGGATAAGAGACCTATAAAT |
|  | ENSDARG00000000966 | nuclear receptor corepressor 2 | ncor2 | CTCTCTCTCTGTCAGAGATACCAGGTAACTGA |
|  | ENSDARG00000091512 | nuclear factor related to kappaB binding protein | nfrkb | TTCAGCTCTAGTACCTTCTCCGTTATAAGAGG |
| Set 15 | ENSDARG00000098272 | NIPBL cohesin loading factor a | nipbla | TGTCTGCTTATTACTGAACTGATGCTATAAAT |
|  | ENSDARG00000061052 | NIPBL cohesin loading factor b | nipblb | AGAGCATGACTGTAACTCTCTGCTTTAACTGA |
|  | ENSDARG00000055022 | PDS5 cohesin associated factor A | pds5a | CTTCTTCATAAAGTCCTGAGCTTTGTAAGAGG |
| Set 16 | ENSDARG00000002971 | polyhomeotic homolog 1 | phc1 | CATAGCAGTAACCATGGTAACAACATATAAAT |
|  | ENSDARG00000008141 | retinoblastoma-like 1 (p107) | rbl1 | CTTGAGTCATTACATTCTCCAGGATTAACTGA |
|  | ENSDARG00000062977 | sterile alpha motif domain containing 13 | samd13 | TCGTTACGAGTCATTAATAAAAGGGTAAGAGG |
| Set 17 | ENSDARG00000102810 | sterile alpha motif domain containing 1a | samd1a | AGATCATCATGGGTAGGAGAGTTCTTATAAAT |
|  | ENSDARG00000057854 | sin3A-associated protein | sap18 | ATTTCTTTAACCCGGTAGATTTGTCTAACTGA |
|  | ENSDARG00000030213 | sap30-like | sap30l | TTCTTGTGGAAGTCACAAATGTAAATAAGAGG |
| Set 18 | ENSDARG00000104311 | Scm polycomb group protein homolog 1 | scmh1 | AGTTTAATGGGATCAGTTGGATTTTTATAAAT |
|  | ENSDARG00000031495 | SET nuclear proto-oncogene a | seta | ATTTCTTTGGAGAGGACTTTGTTCTTAACTGA |
|  | ENSDARG00000003920 | SET nuclear proto-oncogene b | setb | TCAAATGGAACTCTTTTGAGAAAACTAAGAGG |
| Set 19 | ENSDARG00000079716 | SIN3 transcription regulator family member Aa | sin3aa | CAAGGAAGTTCTTAAACCAAGTGAATATAAAT |
|  | ENSDARG00000062472 | SIN3 transcription regulator family member B | sin3b | GTGTTTTCTTGGAACTCACAAAAGTTAACTGA |
|  | ENSDARG00000098919 | SWI/SNF related, matrix associated, actin dependent regulator of chromatin, subfamily c, member 1b | smarcc1b | AATACAGTTGTAGTCGAACCAGGAGTAAGAGG |
| Set 20 | ENSDARG00000019004 | SWI/SNF related, matrix associated, actin dependent regulator of chromatin, subfamily d, member 1 | smarcd1 | AAAAGAAGAAAACTTCCTCTTCTGCTATAAAT |
|  | ENSDARG00000033889 | SPT2 chromatin protein domain containing 1 | spty2d1 | GCAGAAGTTGGAGTTCTTTCACTAGTAACTGA |
|  | ENSDARG00000011783 | stromal antigen 2a | stag2a | ACTGGGTTTTCTTTTCTTTAGCTGTTAAGAGG |
| Set 21 | ENSDARG00000079949 | SPT16 homolog, facilitates chromatin remodeling subunit | supt16h | **CAGATTGGAGCAGTAGGACTTGTAG**TATAAAT |
|  | ENSDARG00000070256 | SUZ12 polycomb repressive complex 2 subunit a | suz12a | **TATCTCCTGCATGGAGACTTCATAT**TAACTGA |
|  | ENSDARG00000098924 | SUZ12 polycomb repressive complex 2 subunit b | suz12b | **GAAGAGTGCTAATTCTGCTCTCTGT**TAAGAGG |
| Set 22 | ENSDARG00000016181 | tripartite motif containing 33 | trim33 | ATACTAATGAGTCTCGGAGGAGGTTTATAAAT |
|  | ENSDARG00000026664 | URI1 prefoldin like chaperone | uri1 | TTTCAGCTCCAATATATATTCCTGCTAACTGA |
|  | ENSDARG00000099273 | zgc:103508 | zgc:103508 | TCTTCATGGTTTTCTTAGGTGACTCTAAGAGG |
| Set 23 | ENSDARG00000062361 | PAX3 and PAX7 binding protein 1 | zgc:158234 (paxbp1) | TCCGACGATTCATCTTTAATATCATTATAAAT |
|  | ENSDARG00000015161 | zgc:92664 | zgc:92664 | TGATGACTTAATGCTAGATGGACCTTAACTGA |
|  | ENSDARG00000054574 | zinc finger, HIT-type containing 1 | znhit1 | TTGGACCCTCACTAACACTTAAGTCTAAGAGG |
